# Effects of Rambutan Peel (*Nepheliumlappaceum*) PhenolicExtract on RANKL-Induced Differentiation of RAW264.7 Cells into Osteoclasts and Retinoic Acid-Induced Osteoporosis in Rats

**DOI:** 10.3390/nu12040883

**Published:** 2020-03-25

**Authors:** Yongliang Zhuang, Xiaodong Sun, Bingtong Liu, Hu Hou, Yun Sun

**Affiliations:** 1 Institute of Agriculture and Food, Kunming University of Science and Technology, No. 727 South Jingming Road, Kunming 650500, China; ylzhuang@kmust.edu.cn (Y.Z.); 15615641901@163.com (X.S.); liubingtong0126@163.com (B.L.); 2 Food Science and Technology, Ocean University of China, No 5, Yushan Road, Qingdao, Shandong 266005, China; houhu@ouc.edu.cn

**Keywords:** rambutanpeel phenolic, osteoporosis, RAW264.7 cells, receptor activator nuclear factor-kappa B ligand(RANKL), histological analysis, bone quality

## Abstract

Previous studies have shown that rambutan peel phenolic (RPP) extract has excellent biological activities due to its abundant phenolic content and profile. In this study, the potential anti-osteoporosis (OP) effects of RPP were evaluated by suppressing receptor activator nuclear factor-kappa B ligand (RANKL)-induced differentiation of RAW264.7 cells into osteoclasts and amelioratingretinoic acid-induced OP in rats. Our results showed that RPP efficiently decreased the formation of tartrate-resistant acid phosphatase (TRAP)-positive cells and reduced total TRAP activity in RAW264.7 cells under RANKL stimulation. RPP treatment significantlyameliorated retinoid acid-induced calcium loss in rats (*p* < 0.05). The serum phosphorus level of osteoporotic rats was increased by RPP treatment, and the serum levels of total alkaline phosphatase and osteocalcin in osteoporotic rats were further reduced. RPP treatment improved the qualities of the femur and tibia, such asbone mineral density, bone length, bone maximum load, cortical bone area ratio, and trabecularelative bone density in osteoporotic rats to some extent. Furthermore, histological analysis showed that RPP effectively improved the bone microstructure of osteoporotic rats by regulating the cortical bone thickness and trabecular bone separation. These results indicate that RPP could have potential applications as a newnutraceutical and functional food in the prevention of OP.

## 1. Introduction

Osteoporosis (OP) is a common bone disease characterized by decreased bone density, increased bone fragility, and increased risk of fracture [1]. According to the World Health Organization report, with the global population aging, approximately 72% of women and 62% of men over 50 years old are estimated to suffer from OP or osteopenia by 2022 [2]. OP has a high incidence of fractures and mortality rates and has become a global epidemic [3]. The bone maintains structural and functional stability through continuous bone remodeling [4]. The balance between bone-forming osteoblasts and bone-resorbing osteoclasts plays a key role in maintaining bone remodeling [5]. Osteoclasts are multinucleated cells formed by the autolysis of monocytes and macrophages and can develop and adhere to bone matrices. The physiological function ofosteoclastsis to degrade the bone matrix by secreting acid and lyase [6]. The bone resorption of osteoclasts is the main reasons that leads bone loss. Osteoclast formation is regulated by specific cytokines. Previous studies have shown that the receptor activator nuclear factor-kappa B ligand (RANKL) is a key cytokine involved in osteoclast differentiation from precursors and activation or survival of osteoclasts [7]. RANKL plays an important part in controlling the function and survival of mature osteoclasts. The interaction between RANKL and its receptor RANK activates several transcription factors [8]. The activation of these factors promotes the expression of genes involved in osteoclast differentiation and function, ultimately differentiating the osteoclast precursor into a preosteoclast [9,10]. Multiple preosteoclasts further fuse together to form huge multinuclear bone-resorbing mature osteoclasts [8,11]. The formation of osteoclasts increases their activity, which leads to an imbalance in bone remodeling [12]. Therefore, the inhibition of osteoclast activity is essential in preventing and treating OP. Currently, many researchers have developed a variety of substances in treating OP, such as bisphosphonates and estrogens, which can induce osteoclast apoptosis as the main target for anti-resorption therapy [13]. However, these substances demonstrate some side effects, including jaw necrosis and high risk of fracture [13]. Thus, finding new potential natural products that can prevent OP effectively and safely is necessary. 

RAW264.7 cells are a well-established preosteoclast cell line. After treatment with specific growth factors, such as RANKL, RAW264.7 cells can be differentiated into osteoclasts [2]. Therefore, RANKL-induced RAW264.7 cells may be used for the study of osteoclasto genesis in vitro. To further systematically study the pharmacological effects of the substanceson OP, the establishment of in vivo animal models of OP is needed. Currently, three animal models of OP, including retinoic acid induction, ovariectomy, and glucocorticoid modeling, are often chosen [14]. Among them, retinoic acid induction has the most simple and rapid operation and highest success rate. In addition, it has better similarity with humans in the onset symptoms, histomorphology, and bone reaction to estrogen [15]. Generally, retinoic acid could reduce estrogen secretion by damaging the ovaries of rats, enhancing the osteophagous activity of osteoclasts, and ultimately leading to high conversion of OP [14,16]. Wei et al. demonstrated the anti-OP effect of naringin on bone damage by retinoic acid-induced osteoporotic rats [17]. 

Phenolic compounds have received considerable attention as important secondary plant metabolites. Phenolic compounds show many potential beneficial effects, including antioxidant [18], antimicrobial [19], hepatoprotective [20], and anti-inflammatory effects [21]. Moreover, many studies have shown that phenolic extracts from different plants, such as tea [22], olives [23], grapes [24], and black beans [25], demonstrate anti-OP properties. Rambutan (*Nepheliumlappaceum*) is a common subtropical fruit species in Southeast Asia [26]. In our previous study, we obtained rambutan peel phenolic (RPP) extract. The phytochemical compounds of RPP extract were further identified and RPP extract is rich in geraniin, procyanidintrimers, procyanidin dimmers, catechin, corilagin, and ellagic acid [27]. Some potent bioactivities of RPP extract were evaluated, including free radical scavenging, anti-photoaging, anti-inflammatory, anti-diabetic as well as anti-aging activities [27,28,29,30]. Furthermore, Zhang et al. found that geraniin can reduce the formation of osteoclasts in vitro and inhibit the bone resorption of osteoclasts [31]. Panahande et al. reported that French maritime pine bark extract, which is rich in procyanidin, has a beneficial effect on bone markers in women with postmenopausal osteopenia [32]. Shalan et al. demonstrated that the catechin-rich extract isolated from noni leaves and black tea can enhance the formation and survival of osteoblasts and inhibit the growth and activity of osteoclasts by using an ovariectomy-induced rat OP model [33]. Rantlha et al. demonstrated that ellagic acid can alleviate osteoclasto genesis by suppressing specific signaling pathways and exerting inhibitory effects on bone resorption [34]. Based on the above findings, RPP may have the potential to inhibitosteoclast formation and anti-OP.

However, few studies have explored whether RPP has an anti-OP effect. The present study aimed to demonstrate the effects of RPP in preventing and treating OP. RANKL-induced RAW264.7 cells were used to assess the effects of RPP on osteoclast formation and differentiation, and there tinoic acid-induced OP modelin rats was used to systematically study the pharmacological effects of RPP.

## 2. Materials and Methods 

### 2.1. Materials and Reagents

RPP samples were obtained according to the previously described method. Briefly, 10 g of dried rambutan peel were microwave-assisted extracted with 245 mL 80.8% (v/v) ethanol for 58 s. The liquid extraction was filtered, concentrated, and freeze-dried to obtain the crude RPP. The crude RPP was purified by NKA-9 resin, which was eluted by 60% (v/v) ethanol [26]. RANKL was purchased from R&D Systems (Minneapolis, MN, USA). 3(4,5-Dimethylthiazol-2-yl)-2,5-diphenyltetrazolium bromide (MTT) and a tartrate-resistant acid phosphatase (TRAP) staining kit(Catalog No: 387A-1KT) were purchased from Sigma-Aldrich (Saint Louis, MO, USA), and a TRAP activity kit(Catalog No: P0332)was obtained from Beyotime Biotechnology (Shanghai, China).Serum calcium(C004-2-1), phosphorus(C006-1-1), alkaline phosphatase (ALP) (TWp003360),and osteocalcin (OCN)(tw042267)assay kits were obtained from Nanjing Jiancheng Bioengineering Institute (Nanjing, China).Serum calcium content was determined by the methylthymol blue colorimetry method, and phosphorus content was determined by the phosphomolybdic acid colorimetry method. Serum ALP and OCN activities were determined by enzyme-linked immunosorbent assay.

### 2.2. Cell Culture

RAW264.7 cells were obtained from Kunming Institute of Zoology (Kunming, China) and cultured in Dulbecco’s modified Eagle’s medium (Thermo Fisher Scientific, Waltham, MA, USA) containing 10% fetal bovine serum (Gibco). Thesolution was mixed with 1% penicillin–streptomycin (Solarbio, Beijing, China). The cells were incubated at 37 °C in a humidified atmosphere of 5% CO_2_.

#### 2.2.1. Cell Viability Assay

Cell viability was measured by MTT assayas described previously [35]. In brief, RAW264.7 cells were cultured in a 96-well plate with a density of 5 × 10^3^ cells/well. Then, the cells were cultured with different concentrations of RPP (0.5, 1.0, 2.5, and 5.0 μg/mL) for 48 h. Thereafter, MTT was added to each well and incubated at 37 °C for 4 h. The medium was then carefully removed. Dimethyl sulfoxide was added to each well, and the optical density was measured at 492 nm using a microplate reader (Spectra Max M5; Molecular Devices, Sunnyvale, CA, USA).

#### 2.2.2. TRAP-Positive Cell Staining 

RAW264.7 cells were cultured in 48-well plates with a density of 5 × 10^3^ cells/well overnight. Five replicates were set for each group of samples. The medium was then replaced with a medium containing 50 ng/mL RANKL and different concentrationsofRPP (1.0, 2.5, and 5.0 μg/mL). The medium and cell factors were replaced every 2 days. After 5 days of culture, TRAP expression of the cells was determined using a TRAP staining kit according to the manufacturer’s instructions. The TRAP-positive cells were observed to be purplish red under an inverted microscope (Olympus, Tokyo, Japan).

#### 2.2.3. Total TRAP Activity

RAW264.7 cells were cultured in the same conditions as the TRAP-positive cell staining assay.Five replicates were set for each group of samples. After 5 days of culture, the medium was carefully removed, and the cell monolayer was gently washed twice with phosphate buffered saline (PBS). The TRAP activity in cells was determined using a TRAP activity kit according to the manufacturer’s instructions. 

### 2.3. Animal Experiments

All animal experiments were conducted in strict accordance with the animal experiment procedures approved by the Animal Care and Use Committee of Kunming University of Science and Technology. Thirty-two specific-pathogen-free Sprague-Dawley (SD) female rats (12 weeks, 220–230 g) were obtained from the Laboratory Animal Center of Jilin University (Jilin, China). All rats were acclimatized for a week in the control environment of the animal room (ambient temperature 20–25 °C, 12 h dark/12 h light cycle) and had free access to standard pellet feed and water. The bedding materials and cages were replaced daily. The drinking bottles were cleaned and refreshed daily. After feeding adaptation for 1 week, eightrats were randomly selected as normal control (NC) group. The NC rats were fed a normal diet, and the other rats were fed with retinoic acid at 75 mg/kg body weight (bw)/day through the intragastric route for 2 weeks. Thereafter, the rats, except the NC group, were randomly divided into three groups, and each group contained eight rats. The three groups were the model group (MC, normal saline taken orally daily), the positive control group (PC, 5 mg/kg bw alendronate taken orally daily), and RPP group (RPP, 20 mg/kg bw RPP taken orally daily). The bw of each rat was monitored to adjust the oral dose, and the data were recorded weekly for 3 weeks. 

#### 2.3.1. Serum Biochemical Assays

The rats underwent fasting overnight at the end of the experiment. All rats were anaesthetized with chloral hydrate, and the blood sample of each rat was collected. The serum was obtained from the blood sample by centrifugation at 2000 r/min for 5 min at 4 °C. The levels of calcium, phosphorus, ALP, and OCN in the serum of rats were determined using relative commercial kits. All experimental procedures were operated strictly in accordance with the kit instructions.

#### 2.3.2. Measurement of Organ Coefficients

The liver of each rat was collected and weighed. The femur and tibia of the rats were collected. The externally adhered tissues, such as meat, fat, and tendons, were removed with a sterile surgical blade and the bone marrow was retained. The left femurs and tibias were dried at 110 °C for 2 h and weighed. The organ coefficients were calculated according to the following formula: organ coefficient = organ weight/body weight × 100.

#### 2.3.3. Bone Quality Assays

The bone quality indices ofthe rightfemur and tibia, including length, bone mineral density (BMD), bone calcium content, and bone maximum load, were analyzed. The length of femurs and tibias was measured with an electronic vernier caliper (DL91150, Deli, China). The BMD of the entire femur and tibia was measured by microcomputed tomography scanner (Latheta LCT-200, Hitachi Aloka Medical, Japan), and the scanning process was set as 70 kV, 80 μA X-ray energy, and 9 μm isotropic voxels. The bone calcium content was measured by flame atomic absorption spectrometry (novAA^®^ 350, Analytikjena, Germany) according to the manufacturer’s instructions. The bone maximum load was evaluated by the three-point bending mechanics experiment using a universal testing machine (CMT5505, MTS industrial Systems, Beijing China). The load was applied to the midpoint of the bone at the calibration 10 kg, loading speed 2 mm/min, span 20 mm until the fracture occurred, and the maximum load of the left femurs and tibias were recorded. The cortical bone area ratio and trabecular relative bone density were analyzed by Leica Qwin image analysis software (Leica, Germany).

#### 2.3.4. Histopathological Assays

The assessment of osteoclast formation in the femur was determined by TRAP staining according to the manufacturer’s instructions (Sigma, 387-A, St. Louis, MO, USA).

Fresh tissue samples were collected rapidly from the right femur, fixed in 10% buffered formalin, and decalcified. Then, the right femur was embedded in paraffin by standard sampling and trimming procedures. The paraffin-embedded tissues were cut into 2–3 µm-thick sections and stained with hematoxylin and eosin (H&E). Histopathological characteristicsin the femur were determined using an Olympus DP80 Digital Camera System (Olympus, Tokyo, Japan).

### 2.4. Statistical Analyses

Data were expressed as means ± standard deviation. The data were analyzed by one-way ANOVA, and Tukey’s procedure was used to determine the significant differences (*p* < 0.05). All analysis was performed on the data using SPSS software package (version 19.0, IBM Inc., Chicago, IL, USA).

## 3. Results

### 3.1. Effect of RPP on Viability of RAW264.7 Cells

**Figure 1:**
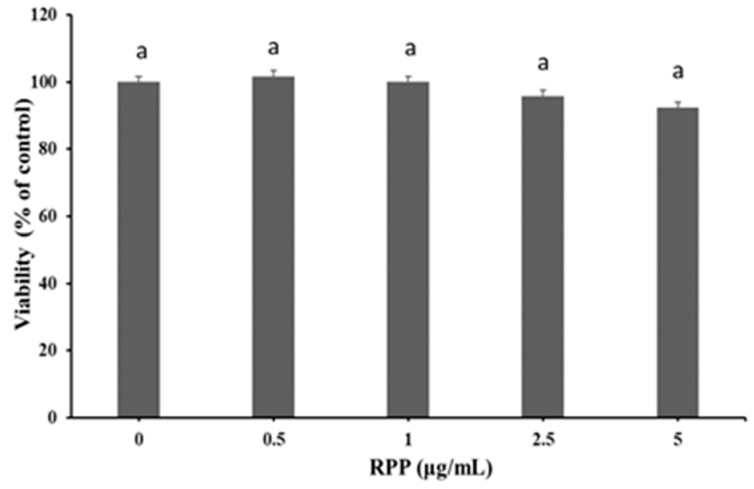
Effect of rambutan peel phenolic (RPP) on the viability of RAW264.7 cells. Groups with different letters are significantly different (*p* < 0.05).

As shown in Figure 1, no significant decrease was observed in cell viability at each RPP concentration (0.5, 1, 2.5, and 5 μg/mL) compared with the control group (*p* > 0.05). Therefore, the bioactivity of RPP was evaluated at concentrations of <5 μg/mL in the following experiments.

### 3.2. Suppressive Effect of RPP on RANKL-Induced Osteoclastogenesis

**Figure 2:**
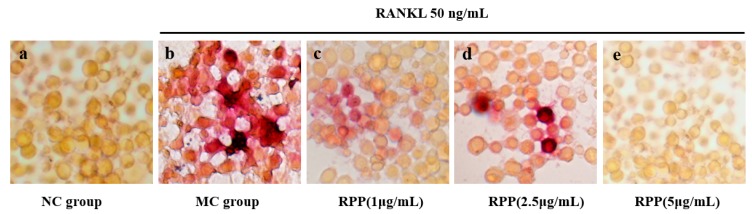
Effects of RPP on receptor activator nuclear factor-kappa B ligand (RANKL)-induced differentiation of RAW264.7 cells into osteoclastcells. (**a**) Normal control (NC) group; (**b**) model group (MC) (RANKL); (**c**) RPP-L (RANKL, 1 μg/mL RPP); (**d**) RPP-M (RANKL, 2.5 μg/mL RPP); (**e**) RPP-H (RANKL, 5 μg/mL RPP). The cells were stained for tartrate-resistant acid phosphatase (TRAP) and the magnification was 400×.

The osteoclast formation was detected using TRAP staining. As shown in Figure 2, RAW264.7 cells in the MC group were differentiated into mature osteoclasts after RANKL stimulation, resulting in a higher purplish red area in the visual field than in the normal control (NC) group. Compared with the MC group, RPP treatment decreased the number of TRAP-positive cells significantly (*p* < 0.05), and the effects were in a dose-dependent manner.

**Figure 3:**
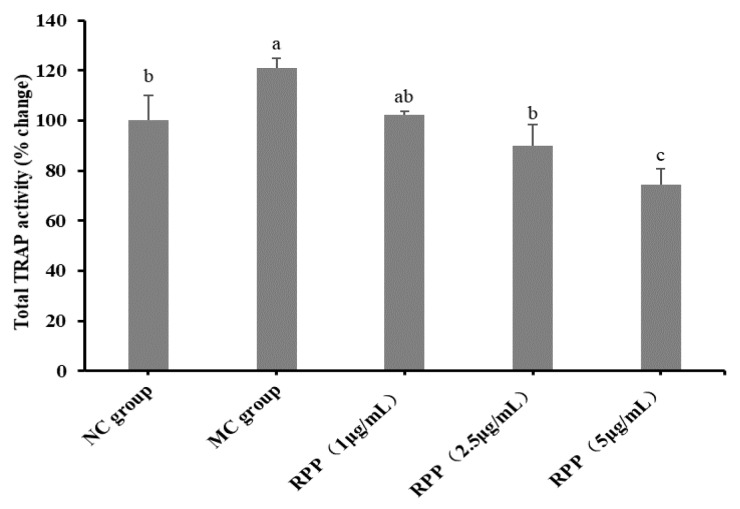
Quantitative assessment of TRAP-positive activity. Groups with different letters are significantly different (*p* < 0.05).

Furthermore, total TRAP activity in RANKL-stimulated RAW264.7 cells was examined. As shown in Figure 3, the total TRAP activity in the MC group was significantly higher than that in the NC group (*p* < 0.05). RPP treatment dose-dependently decreased the total TRAP activity in comparison with the MC group. 

### 3.3. Animal Experiment

#### 3.3.1. Body Weight

**Figure 4:**
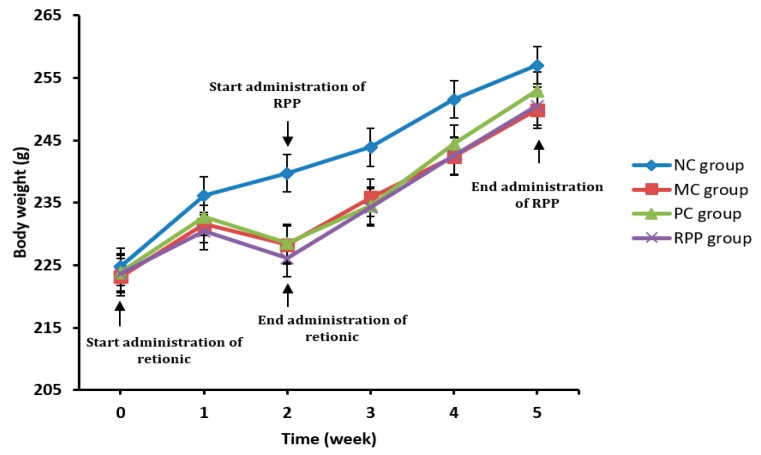
Effect of RPP on body weights of therats in different groups.

As shown in Figure 4, the bw of the rats in the NC group increased gradually with increasing age during the experiment. In the first week of intragastric administration of retinoic acid 75 mg/kg bw, the bw gains of MC, PC, and RPP groups were slow compared with the NC group. In the second week, the bw loss was observed in the MC, PC, and RPP groups, which was consistent with previous studies [36]. In the third week, the bws of these three groups gradually increased but were lower than that in the NC group. No statistical differences of bws in rats were observed between RPP and PC groups (*p* > 0.05).

#### 3.3.2. Serum Biochemical Indicators 

**Figure 5:**
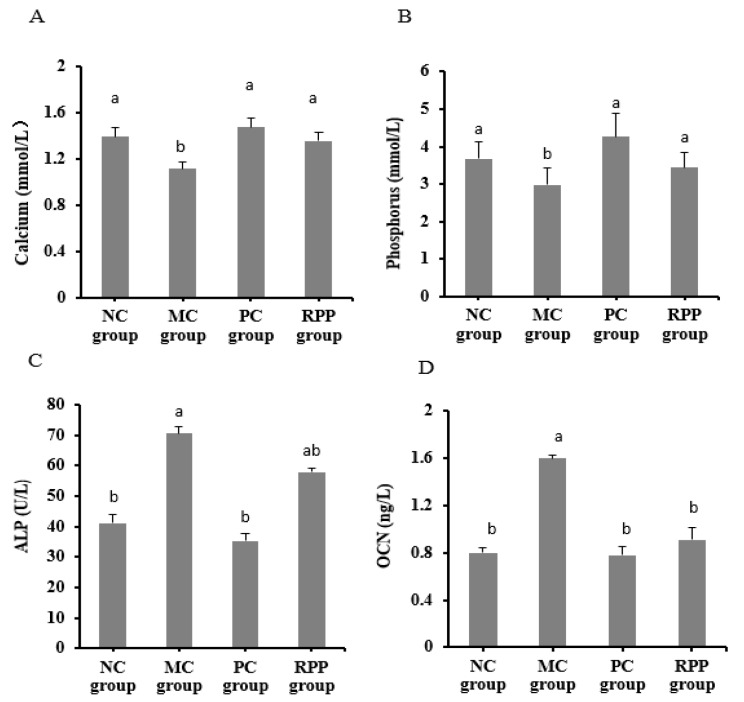
Effect of RPP on blood biochemical parameters in rats. Serum levels of (**A**) calcium, (**B**) phosphorus, (**C**) alkaline phosphatase (ALP), and (**D**) osteocalcin(OCN) of rats in each group were determined. Groups with different letters are significantly different (*p* < 0.05).

Serum biochemical indicators were analyzed, including serum calcium, serum phosphorus, ALP, and OCN, which are widely used in analysis of OP [37]. As shown in Figure 5A,B, the calcium and phosphorus levels in the MC group significantly decreased (*p* < 0.05) compared with the NC group. The serum calcium and phosphorus levels in the PC and RPP groups were significantly higher than those in the MC group (*p* < 0.05). No significant differences were observed in the calcium and phosphorus levels between the RPP and NC groups (*p* > 0.05).

The levels of ALP and OCN were considerably increased in the MC group (*p* < 0.05; Figure 5C,D) compared with the NC group. The level of ALP in the RPP group decreased by17.93%, compared with the MC group. The RPP and PC groups showed a significant reduction in OCN levels (*p* < 0.05). The level of OCN in the RPP group decreased by 43.11%, compared with that in the MC group.

#### 3.3.3. Organ Coefficients 

**Figure 6:**
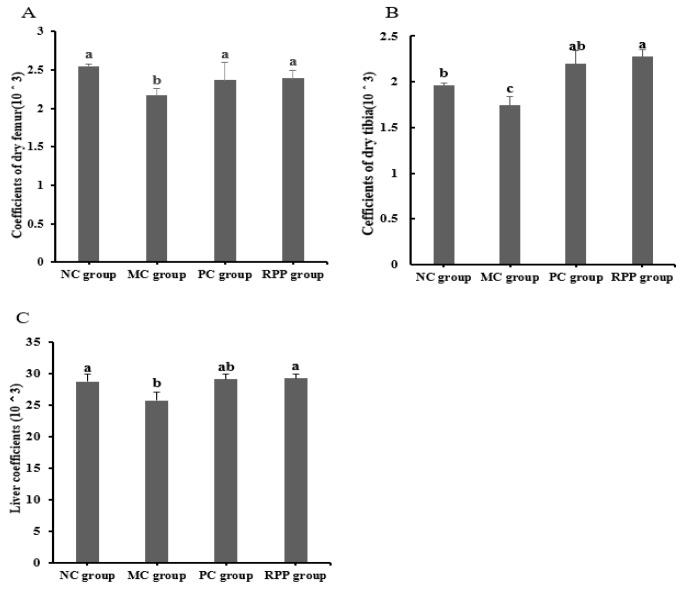
Effect of RPP on organ coefficients in rats. Organ coefficients of(**A**) femur, (**B**) tibia, and (**C**) liver in each group were determined. Groups with different letters are significantly different (*p* < 0.05).

The organ coefficients of the rats in different groups were measured and are shown in Figure 6. The coefficient of the femur in the RPP group had no significant difference with the PC group (*p* > 0.05, Figure 6A), and both coefficients were higher than that in the MC group (*p* < 0.05). The coefficient of the tibia in the MC group was significantly lower than that in the NC group (*p* < 0.05). A significant increase of the tibia coefficient was observed in the RPP treatment group (*p* < 0.05) compared with the MC group, and no significant difference was observed between the RPP and PC groups (*p* > 0.05; Figure 6B). As shown in Figure 6C, the liver coefficient in the RPP group had no significant difference with those of the NC and PC groups (*p* > 0.05).

#### 3.3.4. Bone Quality

**Figure 7:**
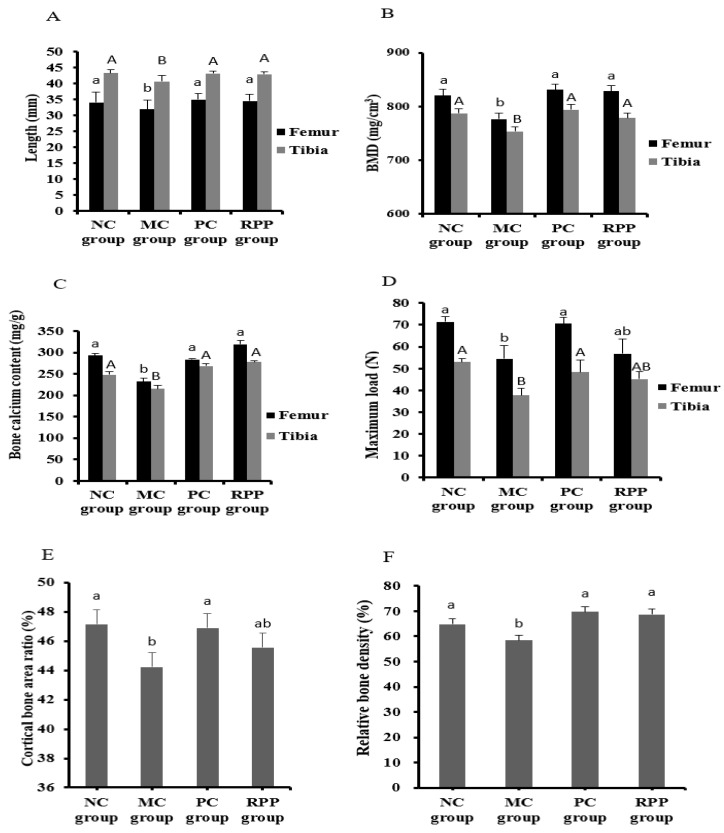
RPP on bone quality in rats. (**A**)Femoral and tibial length, (**B**) bone mineral density (BMD), (**C**) bone calcium content, (**D**) maximum load of femur and tibia, (**E**) cortical bone area ratio, and (**F**) trabecular relative bone densityin each group were determined. Groups with different letters are significantly different (*p* < 0.05).

Bone quality, including the femoral and tibial length, BMD, bone calcium content, and bone biomechanical properties of rats in each group were determined. As shown in Figure 7A, the femoral and tibial length in the MC group were significantly shorter than that in the NC group (*p* < 0.05). No significant difference was observed between the RPP and NC groups in the femoral and tibial length (*p* > 0.05). The BMD of the femur and tibia in the MC group was significantly lower than that of other groups, and no significant differences were observed among the NC, PC, and RPP groups (Figure 7B). As shown in Figure 7C, the bone calcium content in the MC group was significantly decreased compared with that in the NC group (*p* < 0.05). The retinoic acid-induced bone calcium content decrease was markedly prevented by RPP treatment (*p* < 0.05). The maximum load of the femur and tibia significantly decreased in the MC group, compared with the NC group (Figure 7D), while no significant difference was observed between the RPP group and the NC group (*p* > 0.05). Further more, Figure 7E, F reveals the cortical bone area ratio and trabecular relative bone density of the bonesin rats. Both indicators in the MC group were significantly lower than in the NC group (*p* < 0.05), and no significant difference was observed between the RPP and NC groups (*p* > 0.05).

#### 3.3.5. Histopathology

**Figure 8:**
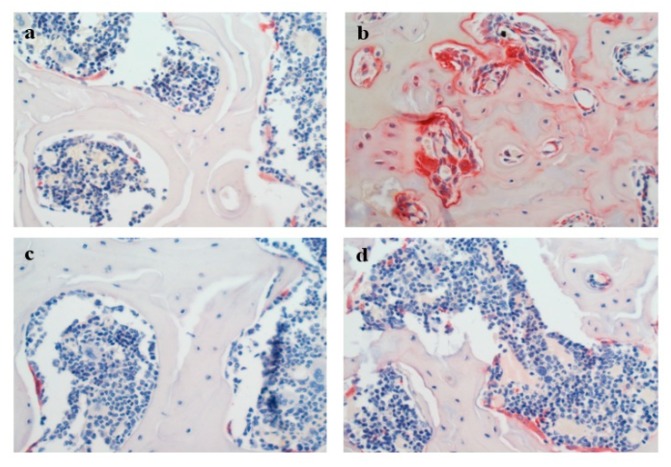
Effect of RPP on TRAP-positive cells formation in bone tissues of rats(magnification 400×). The osteoclasts were stained in red. (**a**) NC group; (**b**) MC group (rats treated with 75 mg/kg retinoid acid); (**c**) positive control (PC) group (retinoid acid treated rats with 5 mg/kg alendronate); (**d**) RPP group (retinoid acid treated rats with 20 mg/kg RPP).

The formation of osteoclasts in the rat femur was determined using TRAP staining. As shown in Figure 8, few TRAP-positive cells were observed in the NC group. Compared with the NC group, the TRAP-positive area was obviously higher in the MC group. The TRAP-positive area in rats treated with RPP was obviously decreased compared with the MC group.

**Figure 9:**
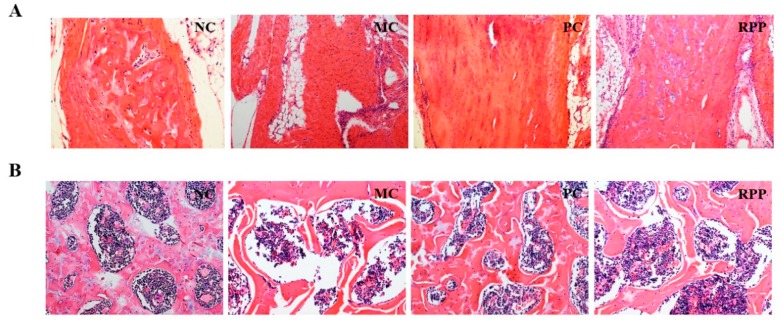
Hematoxylin and Eosin (H&E) staining in right pelvic limb femurs of rats (magnification 400×). The microarchitectures of the (**A**) cortical and (**B**) trabecular bones were detected.

The microarchitectures of the cortical and trabecular bones in the right femur were analyzed by H&E staining. The H&E staining of diaphysis and epiphysis of the femur is shown in Figure 9. Figure 9A shows the H&E staining of the diaphyseal cortical bone of the femur. The MC group had a significant decrease in cortical bone thickness compared with that in the NC group. RPP treatment increased the retinoic acid-induced changes in cortical bone thickness. Figure 9B shows the H&E staining of the epiphyseal trabeculae bone of the femur. The trabecular bone in the MC group was significantly thinned and separated, compared with that in the NC group. RPP treatment could regulate the retinoic acid-induced abnormal changes of the trabecular bone in rats. 

## 4. Discussion

OP has become one of the most challenging orthopedic diseases of the 21st century [1,3]. The treatment methods for OP aim to prevent bone absorption and promote new bone formation. Currently, the substances for the treatment of OP are limited in clinical application due to certain side effects. Some natural alternative substances with a variety of excellent biological activities have aroused people’s attention [1,38,39]. Phenolic compounds are important secondary plant metabolites and have attracted increasing attention because of their good bioactivities [40]. Previous studies have demonstrated that phenolics from various plants have positive effects in inhibiting osteoclasto genesis in vitro and ameliorating OP in vivo [22,23,24,25], which suggests that phenolic extracts can be used as good natural alternatives to improve bone quality and ameliorate OP. Our previous study indicated that RPP is rich in phenolic compounds and 46 compounds are identified in RPP [26]. In addition, it was found that the active components of phenols from RPP, such as geraniin, catechin, corilagin, and ellagic acid, have aprotective effect of anti-OP [31,32,34]. Therefore, the effects of RPP on OP were evaluated using two models in this study. In vitro, the RANKL-induced RAW264.7 cells were used for the study of osteoclasto genesis. In vivo, a retinoic acid-induced OP rat model was developed to investigate the pharmacological effects of RPP on OP. 

The bone is a dynamic tissue and maintaining bone mass homeostasis is a complex process. Striking a balance between the bone formation of osteoblasts and the bone resorption of osteoclasts is needed [10,41,42]. Bone resorption of osteoclasts may play a more important role in bone mass homeostasis [43]. Thus, osteoclasts have become potential therapeutic targets [44]. In this study, we investigated the inhibitory effect of RPP on RANKL-induced osteoclasto genesis of RAW264.7 cells in vitro. The cytotoxicity of RPP on RAW264.7 cells was first detected, and no toxic effect on RAW264.7 cells was observed at the concentration range of RPP in our experiments. TRAP is often used as a primary marker of osteoclast differentiation due to its high expression in osteoclasts [45], and osteoclast formation was detected by TRAP staining in this study. The result showed that RPP dose-dependently reduced the formation of TRAP-positive cells in RANKL-induced RAW264.7 cells. Furthermore, RPP treatment significantly reduced the total TRAP activity in RAW264.7 cells under the condition of RANKL stimulation. Therefore, RPP can effectively regulate the formation process of OP by inhibiting osteoclast differentiation.

RPP can effectively inhibit osteoclasto genesis in vitro, but the effect of RPP on anti-OP in vivo needs to be further studied. Previous research showed that retinoic acid-induced OP in rats can cause a decrease in bw gain in rats. This finding was similar to our results. The bws of the MC, PC, and RPP groups were significantly lower than that in the NC group in the process of building the model. During treatment with RPP, the bws of the rats obviously increased, and the increased rates of the bws of the MC, PC and RPP groups were similar to those in the NC group. In addition, the liver coefficient was measuredin this study. No significant difference was found between RPP group and NC group, which indicated that RPP had no obvious side effects in rats.

Several bone metabolism biomarkers, such as ALP and OCN, are often used for OP assessment [46]. The results showed that the ALP and OCN levels in the MC group were significantly increased, further proving that the OP model induced by retinoic acid was successful. ALP activity is an important indicator related to bone turnover [3]. In our study, RPP treatment appeared to ameliorate ALP and OCN levels, especially OCN levels, which were not significantly different from NC and PC groups. These results indicate that RPP can ameliorate bone metabolism-related blood indicators in OP rats induced by retinoid acid and may have a positive effect on promoting osteogenesis. In addition, calcium and phosphorus are important elements of bone tissue composition, and serum calcium and phosphorus can be used as biomarkers of BMD [11]. The results showed that the MC group had significantly lower serum calcium and phosphorus levels. RPP treatment ameliorated the levels of calcium and phosphorus in the serum of retinoic acid-induced OP rats. RPP could increase the serum calcium level significantly, indicating that RPP may be used as an effective substance to inhibit the calcium loss of OP.

OP is a chronic bone metabolic disease. The bone quality of different groups of rats was further evaluated in the present study. Previous studies have confirmed that the bone calcium content and BMD in retinoic acid-induced rat OP model were reduced [14,15]. In our study, the same decreasing trend of these indicators was observed in the MC group. RPP treatment regulated the decrease of bone calcium content and BMD in a rat OP model, indicating that RPP can inhibit bone loss in osteoporotic rats induced by retinoic acid. This result was consistent with the increase of serum calciumin RPP group (Figure 5A). Furthermore, RPP treatment increased the coefficients and lengths of femursand tibiasin OP rats compared with the MC group, and the biomechanical properties of the femurs and tibias in the RPP treatment group were also significantly strengthened. In addition, RPP also regulated the cortical bone area ratio and trabecular relative bone density. These results indicate that RPP plays an active role in enhancing bone quality. 

Histopathologic studies, including TRAP and H&E staining, were done to intuitively illustrate the effect of RPP on retinoic acid-induced OP. Our results showed that the TRAP-positive area in bone tissue was significantly reduced under RPP treatment, indicating that RPP treatment can inhibit retinoic acid-stimulated osteoclast formation, thereby reducing the bone resorption capacity of osteoclasts. This result was consistent with the result of TRAP-positive cell staining in RANKL-induced RAW264.7 cells (Figure 2). Bone strength depends not only on the amount of bone mineral content but also on the internal microstructure of the bone [47]. For instance, lower cortical bone thickness and smaller trabecular bone density are believed to be associated with larger bone loss and decreased bone strength, which weaken bone resistance to external forces and easily lead to fractures [47]. In the present study, the microstructures of the femurs of rats were measured by histology. H&E staining showed that the cortical bone thickness was decreased and the trabecular bone was separated in the MC group. These abnormal changes can be regulated in the RPP group. The above results strongly suggest that RPP can ameliorate bone quality, improve bone strength, and further prevent bone deterioration caused by OP.

## 5. Conclusions

The present study demonstrated the anti-OP effect of RPP through two models. In vitro, the RANKL-induced RAW264.7 cell model was used, and RPP effectively decreased osteoclast formation and inhibited osteoclast differentiation. In vivo, a retinoic acid-induced OP rat model was established to evaluate the inhibitory OP ability by the serum biochemical indicators and the physicochemical and histological properties. Our results indicate that RPP can effectively ameliorate retinoid acid-induced OP in rats. Therefore, RPP may have the potential to be exploited as a natural substance for OP treatment.
